# Association of DNA methylation of vitamin D metabolic pathway related genes with colorectal cancer risk

**DOI:** 10.1186/s13148-023-01555-0

**Published:** 2023-08-29

**Authors:** Yi-Fan Wang, Lei Li, Xue-Qing Deng, Yu-Jing Fang, Cai-Xia Zhang

**Affiliations:** 1 Department of Epidemiology, School of Public Health, Sun Yat-Sen University, Guangzhou, 510080 China; 2 Experimental Teaching Center, School of Public Health, Sun Yat-Sen University, Guangzhou, 510080 China; 3 Department of Experimental Research, Sun Yat-Sen University Cancer Center, State Key Laboratory of Oncology in South China, Collaborative Innovation Center for Cancer Medicine, 651 Dongfeng Road East, Guangzhou, 510060 China

**Keywords:** Vitamin D metabolic pathway related genes, DNA methylation, *VDR*, *CYP24A1*, *CYP27B1*, *CYP2R1*, Colorectal cancer

## Abstract

**Background:**

Vitamin D might have anti-tumor effect, which is affected by the genes related to vitamin D metabolic pathway. Epigenetic mechanism may affect the expression level of vitamin D metabolic pathway related genes, then plays an important role in the occurrence and development of colorectal cancer. To date, no study has reported on the association between blood-based DNA methylation level of vitamin D metabolic pathway related genes and colorectal cancer risk.

**Methods:**

A case–control study was conducted including 102 colorectal cancer cases and 102 sex- and age-frequency-matched controls in Guangzhou, China. CpG islands in the *VDR, CYP24A1*, *CYP27B1* and *CYP2R1* genes were chosen for DNA methylation analysis by MethylTarget sequencing. The receiver operating characteristic (ROC) curve was used to evaluate the diagnostic value of DNA methylation levels for colorectal cancer. Taking the point with the largest Youden index as the boundary value, the cumulative methylation levels of vitamin D metabolic pathway related genes were divided into hypomethylation and hypermethylation. Unconditional multivariable logistical regression model was used to calculate the adjusted odds ratio (aOR) and 95% confidence intervals (95% CIs) after adjusting for potential confounders.

**Results:**

Among 153 CpG sites, 8 CpG sites were significantly different between the cases and the controls. The cumulative methylation level of all CpG sites in *CYP2R1* was inversely associated with the risk of colorectal cancer (aOR, 0.49; 95% CI, 0.26–0.91). However, no significant association was found between cumulative methylation levels of all CpG sites in *VDR*, *CYP24A1* and *CYP27B1* and colorectal cancer risk. Significant inverse association was observed between cumulative methylation level of significant CpG sites in *VDR* (aOR, 0.28; 95% CI, 0.16–0.51) and *CYP24A1* (aOR, 0.19; 95% CI, 0.09–0.40) and colorectal cancer risk. There were no significant associations between cumulative methylation levels of significant CpG sites in *CYP2R1* and *CYP27B1* and colorectal cancer risk.

**Conclusions:**

This study indicated that the cumulative methylation levels of significant CpG sites in *VDR* and *CYP24A1* and all CpG sites in *CYP2R1* were inversely associated with colorectal cancer risk.

## Background

Colorectal cancer is the third most common cancer in both sexes and the second leading cause of cancer-related death around the world [1]. In 2020, there were approximately 1.9 million new colorectal cancer and 0.9 million deaths worldwide [1]. In the last two decades, the incidence and mortality of colorectal cancer had been rising in China [2]. More than 555 thousand new colorectal cancer cases and 286 thousand colorectal cancer-related deaths occurred in China in 2020 [3].

The occurrence of colorectal cancer is a complex and multifactorial process. Studies have explored the potential role for vitamin D in the prevention of cancer since Garland et al. hypothesized that increased sunlight exposure and vitamin D were associated with a lower risk of colorectal cancer in 1980 [4]. So far, many epidemiological studies have investigated the association of vitamin D including dietary intake and serum concentration with colorectal cancer risk and the results are mixed [5, 6]. Some previous studies [7, 8], including ours [9], found that dietary vitamin D intake was inversely associated with colorectal cancer risk. Vitamin D must undergo two steps of hydroxylation in the liver and the kidney before it is converted into an active metabolite 1,25-dihydroxyvitamin D (1,25(OH)_2_D). This process is affected by the genes related to vitamin D metabolic pathway such as vitamin D synthesis gene *CYP2R1* and *CYP27B1*, vitamin D metabolic gene *CYP24A1* and vitamin D receptor gene *VDR* [10].

Vitamin D contributes to the occurrence and development of colorectal cancer through genetic and epigenetic effects [11]. While previous studies concentrated on vitamin D metabolism pathway gene polymorphisms [12, 13], more recent studies have investigated the epigenetic modifications related to vitamin D [11]. Epigenetic modifications regulate gene expression without the alternation of DNA sequence [14]. DNA methylation, a transfer of methyl group from S-adenosine methionine to cytosine and forms methylated cytosine in a CpG dinucleotide, is one of the most common epigenetic modifications in the development of cancer [15]. All vitamin D metabolic pathway-related genes have long CpG islands, for example, the promoter regions of the *VDR*, *CYP2R1* and *CYP24A1* genes and the first exon region of the *CYP27B1* gene, which can be gene-silenced by hypermethylation [16]. In addition, studies of specific genes found that the methylation level of vitamin D metabolic pathway genes was associated with vitamin D levels [17, 18]. Beckett et al. examining the methylation status of vitamin D metabolic pathway gene *CYP2R1* in adults over 65 years of age found that 25-hydroxyvitamin D (25(OH)D) was negatively associated with the methylation level of *CYP2R1* [17]. One case–control study demonstrated that patients with severe vitamin D deficiency had higher methylation level of *CYP2R1* and lower methylation level of *CYP24A1* compared to the controls [18].

One previous study has examined the association between methylation levels of vitamin D metabolic pathway related genes and type 2 diabetes mellitus [19]. Some studies have investigated the differences in methylation levels of genes related to vitamin D metabolic pathways in patients with tuberculosis [20] and rheumatoid arthritis [21] compared to healthy individuals. One study explored the difference of the methylation frequency of *VDR*, one of the vitamin D metabolic pathway-related genes, between tumor and normal tissue in colorectal cancer cases [22]. However, no study has been reported on the association between blood-based DNA methylation levels of multiple vitamin D metabolic pathway related genes and colorectal cancer risk systematically. In this context, we conducted this study to examine the association between methylation levels of four vitamin D metabolic pathway-related genes (*VDR*, *CYP24A1*, *CYP27B1* and *CYP2R1*) from blood and the risk of colorectal cancer. We hypothesized that the methylation level of *CYP24A1* could be negatively associated with colorectal cancer risk, while the methylation levels of *VDR, CYP2R1* and *CYP27B1* could be positively associated with colorectal cancer risk.

## Methods

### Study subjects

Data for this study were collected from an ongoing large case–control study launched in July 2010, the details of which were fully outlined previously [23]. The inclusion criteria were that the patients were aged from 30 to 75 years old, had a histological diagnosis of colorectal cancer less than 3 months before the interview, and were Guangdong natives or having stayed in Guangdong Province for 5 years or more. Patients were excluded if they had a history of other cancers, refused to be investigated, or had communication and cognitive impairments. Basically, 3174 cases who met the criteria were recruited from Sun Yat-sen University Cancer Center, Guangzhou, China. Among them, 2833 cases were successfully investigated, and their peripheral blood samples were obtained, with a response rate of 89.26%. Additionally, 27 cases who had unreasonable daily energy consumptions were excluded from the study (< 800 or > 4200 kcal/d for men, < 600 or > 3500 kcal/d for women). Out of 2806 cases with both complete questionnaire information and DNA samples that met the conditions for methylation testing, 102 cases were randomly selected in the present study. The randomization procedure involved generating a random number of the cases by sex and selecting cases in descending order of random number. Finally, 58 male and 44 female cases were included in the analysis, with the sex ratio close to the whole case group.

The inclusion and exclusion criteria for the controls were the same as those for the cases, other than that they had no history of any cancers. Frequency-matched controls were identified based on sex and age at interview (± 5 years). Hospital-derived control group was recruited from the inpatients admitted to the Departments of Vascular Surgery, Plastic Surgery and Otolaryngology of the First Affiliated Hospital of Sun Yat-sen University and the Affiliated Eye Hospital of Sun Yat-sen University during the same time period. Specifically, they were required to be free of illnesses associated with dietary cause. Another control group was recruited from the community residents of the cases' cities via community advertisements, written invitations and recommendations. From July 2010 to May 2021, 1504 hospital-derived controls and 1302 community-derived controls were successfully interviewed. Among controls with qualifying DNA samples, 102 controls were randomly selected and were frequency-matched to cases by sex and 5-year age group.

This study was conducted in accordance with the ethical standards outlined in the 1964 Declaration of Helsinki and its subsequent amendments. The Ethics Committee of the School of Public Health, Sun Yat-sen University approved the study (No: 2019–105). All subjects signed an informed consent form before the interview.

### Data collection

Face-to-face interviews were conducted using a structured questionnaire by trained interviewers. The questionnaire included basic characteristics (e.g., sex, age, occupation, educational level, household income), lifestyle factors (e.g., smoking history, alcohol consumption, physical activity), and the history of disease and first-degree relatives with cancer. Regular smoking was defined as smoking at least one cigarette per day for six months or more [24]. Regular drinking was defined as drinking alcohol at least once a week for more than six months in the past year; we inquired about the type of wine consumed as well as the frequency and amount (Liang) of wine consumed per month over the past year [25]. Body mass index (BMI) is computed by dividing a person's weight in kilograms by the squared height in meters. Metabolic equivalent (MET)-hours/week is obtained by multiplying the average hours per week spent on a particular activity during the past year with the MET score for that particular activity, as in previous studies [26, 27]. A validated 81-food item food frequency questionnaire was used to collect information on dietary intake, including 12 kinds of cereals, 7 kinds of legumes, 18 kinds of vegetables, 11 kinds of fruits, 18 kinds of meat, 2 kinds of eggs, 8 kinds of dairy products, 3 kinds of beverages and soups and 2 kinds of mushrooms and nuts [28]. The frequency and portion size of food intake were required to report within 1 year prior to diagnosis for the cases and enrollment for the controls. Intakes of energy, dietary fiber, vitamin D and other nutrients were estimated according to the China Food Composition Table 2002 [29]. The average daily intake of each nutrient was calculated by multiplying the portion size of each food consumed, the frequency of food intake and the nutrient content of each food.

### Blood sample collection and genomic DNA extraction

Five milliliters overnight fast venous blood samples were drawn from participants on the morning of the second day after admission without any treatment. After collection, blood was centrifuged at 3000 rpm for 15 min at 4 °C. Then, the white blood cells were divided into EP tubes for DNA extraction, and all blood samples were stored at − 80℃ until use.

The Genomic DNA extraction kit (TIANGEN Biotech, Beijing, China) was used to isolate DNA following the manufacturer's instructions. Quality control was performed using NanoDrop ND-2000 and agarose gel electrophoresis. DNA samples were tested for concentration and purity with the Nanodrop ND-2000 apparatus. Agarose gel electrophoresis showed whether the main bands were clear and whether there was significant dispersion or trailing. Only DNA samples with integrity and an A260/A280 within 1.8–2.0 which passed quality inspection were eligible for the subsequent analysis. Then, we used the EZ DNA Methylation Gold™ Kit (Zymo Research Corporation, CA, USA) to proceed bisulfite-conversion.

### Selection of candidate CpG sites

The parameters of CpG islands on four vitamin D metabolism-related genes *VDR, CYP24A1, CYP27B1* and *CYP2R1* were evaluated according to the following criteria: (1) length > 200 bp; (2) GC content > 50%; (3) CpG observation expectation ratio > 0.6. The presence of 2 CpG islands in *VDR*, 4 CpG islands in *CYP24A1*, 1 CpG island in *CYP27B1*, and 2 CpG islands in *CYP2R1* was evaluated, containing 153 CpG sites. Details of each island, including the relative distance to the transcription start site, length, are shown in Table 1.

**Table 1:** Details of the CpG islands of *VDR*, *CYP24A1*, *CYP27B1* and *CYP2R1*

Gene	Fragment	TSS	Start	End	Length	Target Strand	Distance 2TSS	Number of CpG sites
*VDR*	*VDR*_1	47,904,994	47,905,397	47,905,564	168	+	− 403	15
	*VDR*_2	47,904,994	47,904,776	47,905,043	268	+	218	22
*CYP24A1*	*CYP24A1*_1	54,173,986	54,174,469	54,174,217	253	−	− 483	29
	*CYP24A1*_2	54,173,986	54,173,595	54,173,365	231	−	391	17
	*CYP24A1*_3	54,173,986	54,173,176	54,173,013	164	−	810	12
	*CYP24A1*_4	54,173,986	54,173,039	54,172,868	172	−	947	13
*CYP27B1*	*CYP27B1*	57,767,078	57,766,171	57,765,967	205	−	907	17
*CYP2R1*	*CYP2R1*_1	14,892,205	14,891,629	14,891,787	159	+	576	15
	*CYP2R1*_2	14,892,205	14,891,347	14,891,079	269	−	858	13

Start, The starting position of the product on the reference genome; TSS, The mRNA transcription start site; End, The end position of the product on the reference genome; Length, the product length; Target strand, The product orientation; Distance 2TSS, The distance from the product to the TSS

### DNA methylation analysis

The DNA methylation level was attained by MethylTarget assays, a method that enabled multiple CpG islands to be sequenced simultaneously based on a second-generation sequencing platform and was performed by Genesky BioTech (Shanghai, China). Based on the DNA samples treated with bisulfite, the primers were designed and optimized for multiplex polymerase chain reaction (PCR) using Methylation Fast Target software. The optimized primers were mixed into a multiplex PCR primer panel, as shown in Table 2.

**Table 2:** Primer sequences for PCR amplification

Primer name	Sequence
*VDR_1F*	TATTTGGGTTGATTAGGTTAGGATTT
*VDR_1R*	CCCTAATCTATAAAATCAAACTAAACTTCCTAAC
*VDR_2F*	TTAGTGTTTTTTAGTGTTTTAGTTTTATGGTA
*VDR_2R*	ACTAAACTATCTCTACTTATCAAAAAACRACA
*CYP24A1_1F*	AGGTTGGGGGTATTTGGTTTTT
*CYP24A1_1R*	AACTCCACCCCRAAAATAACC
*CYP24A1_2F*	GGTGTTTTTYGTTGTTATGAGTTTTT
*CYP24A1_2R*	TACAACAAACTACCCAACAATAACC
*CYP24A1_3F*	GAGGYGGGAGGAGGGAAAG
*CYP24A1_3R*	CAACAAACATAACRAACCCAAATACA
*CYP24A1_4F*	GTGTATTTGGGTTYGTTATGTTTGTT
*CYP24A1_4R*	CAAATCTAACCRCATACCCAAATC
*CYP27B1_F*	GGTGTGGTTAGTTAGTTTTGGGATAG
*CYP27B1_R*	CTACAAAACRTCTAAACTTCTAAAAACAAAA
*CYP2R1_1F*	TGAGGGTATGYGTTTATTTTGGATTT
*CYP2R1_1R*	AAAAACCCCRCCCCTACC
*CYP2R1_2F*	TGGTTGGGAAYGGTATTTTAGTAG
*CYP2R1_2R*	TCAAAACAAAACAAATAAACTCTATCC

Multiplex PCR was carried out with the primer panel above. Each reaction used a 20-μL PCR reaction system of 10 × buffer (TAKARA), 2.5 mM dNTP, 25 mM Mg^2+^, 1 μM multiplex PCR panel primers, 5U HotTaq polymerase, 1 μL of the bisulfite-treated DNA sample and 11.1 μL of ddH_2_O. The cycle schedules set as follows: 95℃ for 120 s; 11 cycles at 95℃ for 20 s, 60℃ for 30 s with the temperature decreased by 0.5℃ each cycle, 60℃ for 30 s; then 24 cycles at 95℃ for 20 s, 62℃ for 30 s, 72℃ for 1 min, lastly 72 ℃ for 60 s.

The multiplex PCR products of each panel from the same sample were quantitatively mixed in equal proportions. After adding specific labels to each sample, different samples were mixed as well. Besides, PCR amplification products were purified with the Agarose Gel Extraction Kit (TIANGEN Biotech, Beijing, China) after agarose electrophoresis separated. The molar concentrations of the libraries from diverse samples were quality tested and accurately quantified. High-throughput sequencing was carried out using the Illumina Hiseq platform (California, USA) in 2 × 150 bp double-ended mode. After sequencing, Fast QC software was used to assess the quality of data.

### Statistical analysis

Kolmogorov–Smirnov was used for testing the normality of data. The daily intakes of foods and vitamin D were logarithmically transformed, and a residual method was used to adjust the energy [30]. Differences in demographic variables and potential risk factors between the cases and the controls were compared using *t* test (for normally distributed continuous variables) or chi-square test (for categoric variables); the Wilcoxon rank sum test was used to compare the differences in methylation rates of individual CpG site between the case and control groups. CpG sites with statistical differences between the case and control groups were defined as significant CpG sites.

We analyzed both individual and cumulative methylation levels of the candidate genes. Two different models were constructed to further analyze cumulative methylation levels of vitamin D metabolic pathway related genes and their associations with colorectal cancer risk. In Model 1, the methylation rates of all CpG sites in each vitamin D metabolic pathway related gene were summed to obtain the cumulative methylation rates of all CpG sites. In Model 2, the methylation rates of significant CpG sites with the same direction in each gene were summed to obtain the cumulative methylation rates of significant CpG sites.

The receiver operating characteristic (ROC) curve was used to assess the predictive value of colorectal cancer by cumulative methylation rate at all sites and significant sites. The area under the curve (AUC) was calculated. The target gene's methylation status (hypermethylation or hypomethylation) was classified according to the optimal cutoff value, defined as the cumulative methylation rate with the highest Youden index. An unconditional logistic regression model was used to calculate the odds ratio (OR) and 95% confidence interval (CI) to determine the association between methylation status of gene in the vitamin D metabolic pathway and colorectal cancer risk after adjusting for potential confounders. Potential confounders included in the multivariable model were selected based on comparison of characteristics between cases and controls.

A sex-stratified analysis was performed to investigate whether sex made an impact on the association between cumulative DNA methylation levels and colorectal cancer risk. The value of *P*_interaction_ was calculated by placing the multiplication terms of sex and cumulative DNA methylation levels into the regression models. A subgroup analysis was conducted based on the cancer site (colon or rectum) of colorectal cancer patients, and the value of *P*_heterogeneity_ was based on cases only. SPSS 23.0 (IBM Corp, Armonk, NY, USA) was used to complete data processing and analysis. *p* values were two-sided, and *P* < 0.05 was considered a statistically significant difference. We had greater than 95% power to detect OR of 0.28 and 0.19 for the association of cumulative methylation status of *VDR* and *CYP24A1* at significant CpG sites with colorectal cancer risk. Our sample gave us 57% power to detect the OR of 0.49 for the association of cumulative methylation status of *CYP2R1* at all CpG sites with colorectal cancer risk at *P* < 0.05 (two-sided).

## Results

### Characteristics of study subjects

As shown in Table 3, the average age (SD) was 58 (10) years in cases and 57 (9) years in controls. Compared to the controls, the cases had increased numbers of first-degree relatives with cancer (19% vs. 7%) and a lower intake of dietary vitamin D (206 IU/day *vs*. 238 IU/day). There were no significant differences in marital status, residence, educational level, occupation, family income, BMI, smoking, drinking, physical labor at work and dietary intake between the two groups (*P* > 0.05). The variable of first-degree relatives with cancer was considered potential confounder and adjusted for in subsequent analyses.

**Table 3:** Characteristics and dietary intakes of study subjects

Characteristics	Cases (*n* = 102)	Controls (*n* = 102)	*P*
Age (years) (Mean ± SD)	58 ± 10	57 ± 9	0.78
Male (*n*, %)	58 (57)	58 (57)	1
Urban (*n*, %)	72 (71)	73 (72)	0.88
Married (*n*, %)	100 (98)	97 (95)	0.25
Educational level (*n*, %)			0.95
Primary school or below	32 (31)	30 (29)	
Secondary school	27 (26)	25 (25)	
High School	22 (22)	23 (23)	
College or above	21 (21)	24 (24)	
Occupation (*n*, %)			0.74
Administrator/other white-collar workers	20 (20)	17 (17)	
Blue-collar worker	21 (21)	25 (25)	
Farmer/others	61 (60)	60 (59)	
Household income (Yuan/month) (*n*, %)			0.19
< 2000	16 (16)	8 (8)	
2001–5000	25 (25)	36 (35)	
5001–8000	36 (35)	34 (33)	
≥ 8001	25 (25)	24 (24)	
BMI (kg/m^2^) (Mean ± SD)	23 ± 3	24 ± 3	0.42
First-degree relatives with cancer (*n*, %)	19 (19)	7 (7)	0.01
Smoking (*n*, %)	41 (40)	36 (35)	0.47
Regular drinking (*n*, %)	17 (17)	20 (20)	0.59
Occupational activity (*n*, %)			0.85
Non-working	22 (22)	18 (18)	
Sedentary	30 (29)	30 (29)	
Light	25 (25)	25 (25)	
Moderate	14 (14)	13 (13)	
Heavy	11 (11)	16 (16)	
Household and leisure activity (MET-h/week) Median (P_25_, P_75_)	26 (7.5, 52)	26 (7.3, 52)	0.80
Total energy intake (kcal/day) Median (P_25_, P_75_)	1510 (1140, 1750)	1420 (1200,1660)	0.80
Dietary fiber intake (g/day) Median (P_25_, P_75_)	8.8 (6.8, 10)	8.7 (7.4, 10)	0.70
Red and processed meat intake (g/day) Median (P_25_, P_75_)	103 (68, 150)	107 (84, 138)	0.58
Vegetables intake (g/day) Median (P_25_, P_75_)	403 (289, 531)	429 (316, 530)	0.32
Fish intake (g/day) Median (P_25_, P_75_)	93 (40, 144)	106 (52, 164)	0.21
Dietary Vitamin D intake (IU/day) Median (P_25_, P_75_)	206 (79, 408)	238 (126, 502)	0.006

### DNA methylation levels at individual CpG sites

Among 153 CpG sites, 8 CpG sites were significantly different between colorectal cancer cases and controls (*P* < 0.05) (Table 4). Among them, 3 CpG sites were in the *VDR*, 4 CpG sites were in the *CYP24A1* gene, and 1 CpG site was in the *CYP2R1* gene. Except for CpG site of 53, methylation levels of other CpG sites were significantly lower in cases than those in controls.

**Table 4:** Methylation rates of significant CpG sites between the cases and the controls

CpG site	Gene	Position	Cases (*n* = 102) Mean ± SD	Controls (*n* = 102) Mean ± SD	*P*
58	*VDR*	12: 47,905,454	0.018 ± 0.013	0.020 ± 0.008	0.02
106	*VDR*	12: 47,905,502	0.038 ± 0.017	0.040 ± 0.012	0.02
110	*VDR*	12: 47,905,506	0.012 ± 0.008	0.014 ± 0.007	0.02
102	*CYP24A1*	20: 54,174,368	0.018 ± 0.011	0.021 ± 0.011	0.03
200	*CYP24A1*	20: 54,174,270	0.011 ± 0.009	0.014 ± 0.009	0.007
41	*CYP24A1*	20: 54,173,555	0.022 ± 0.008	0.023 ± 0.007	0.047
53	*CYP24A1*	20: 54,172,987	0.050 ± 0.024	0.040 ± 0.011	0.02
90	*CYP2R1*	11: 14,891,718	0.017 ± 0.005	0.019 ± 0.005	0.04

The data of methylation rates of significant CpG sites are expressed as Mean ± SD

### Cumulative DNA methylation levels of multiple CpG sites among colorectal cancer cases and controls

As shown in Table 5, in Model 1, the methylation rates of all CpG sites in each gene were summed up. The cumulative methylation rates of *VDR*, *CYP24A1*, *CYP27B1*, and *CYP2R1* genes were not statistically significant between the case and control groups. In Model 2, we summed the methylation rates of significant CpG sites with consistent direction in each gene. Therefore, CpG site 53 in the *CYP24A1* gene was excluded that was more frequently methylated in colorectal cancer cases than in controls. The *CYP2R1* was not included because it had only one significant CpG site. Finally, a total of six hypomethylated CpG sites in the *VDR* and *CYP24A1* genes were included. The cumulative methylation levels of the *VDR* and *CYP24A1* were significantly lower in colorectal cancer cases than those in controls (*P* < 0.05).

**Table 5:** Comparison of cumulative methylation rates at all CpG sites and significant CpG sites

Gene	Cases (*n* = 102) Mean ± SD	Controls (*n* = 102) Mean ± SD	*Z*	*P*
Model 1 (all CpG sites)				
*VDR*	0.46 ± 0.11	0.46±0.07	− 1.45	0.15
*CYP24A1*	1.85 ± 0.45	1.83±0.34	− 0.32	0.75
*CYP27B1*	0.54 ± 0.18	0.54±0.14	− 0.58	0.56
*CYP2R1*	0.28 ± 0.04	0.30±0.16	− 0.76	0.45
Model 2 (significant CpG sites)				
*VDR*	0.06 ± 0.03	0.07 ± 0.02	− 3.58	0.001
*CYP24A1*	0.05 ± 0.02	0.06 ± 0.02	− 3.25	< 0.001

The data of cumulative methylation rates at all CpG sites and significant CpG sites are expressed as Mean ± SD

### The diagnostic value for colorectal cancer of cumulative methylation levels using different models

The values of cumulative methylation rates of vitamin D metabolic pathway related genes in the diagnosis of colorectal cancer are shown in Table 6. In Model 1, the cumulative methylation level of the *VDR* gene at all CpG sites showed the highest diagnostic value with an AUC of 0.56 (95% CI, 0.48–0.64), followed by the *CYP2R1* gene with an AUC of 0.53 (95% CI, 0.45–0.61).

**Table 6:** Diagnostic value for colorectal cancer of cumulative methylation levels

Gene	Model 1				Model 2		
	all CpG sites				significant CpG sites		
	AUC	95% CI	*P*		AUC	95% CI	*P*
*VDR*	0.56	0.48–0.64	0.15		0.65	0.57–0.72	0.001
*CYP24A1*	0.51	0.43–0.59	0.75		0.63	0.56–0.71	< 0.001
*CYP27B1*	0.52	0.44–0.60	0.56		–	–	–
*CYP2R1*	0.53	0.45–0.61	0.45		–	–	–

In Model 2, the value of cumulative methylation of *VDR* and *CYP24A1* at significant CpG sites in the diagnosis of colorectal cancer was evaluated. The AUC of *VDR* and *CYP24A1* were 0.65 (95% CI, 0.57–0.72) and 0.63 (95% CI, 0.56–0.71), respectively. The AUCs of both *VDR* and *CYP24A1* in Model 2 were higher compared to these in Model 1.

### Association of cumulative methylation levels at all CpG sites of genes in vitamin D metabolic pathway with colorectal cancer risk

**Fig. 1:**
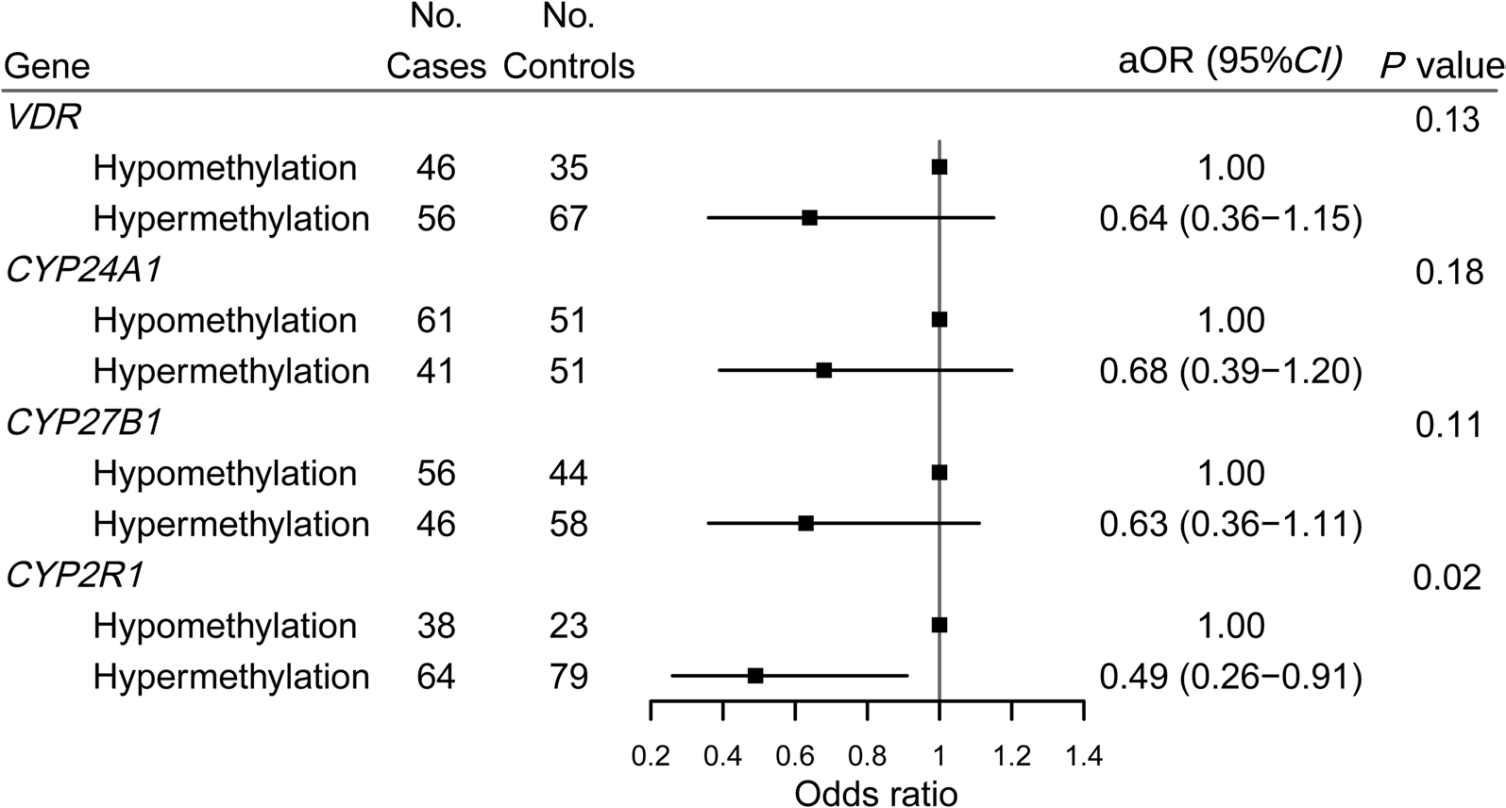
Association between cumulative methylation status at all CpG sites of the vitamin D metabolic pathway genes and colorectal cancer risk. OR was adjusted for the first-degree relatives with cancer

The methylation level at all CpG sites of individual gene was classified as hypermethylation or hypomethylation according to the cumulative methylation rate with the highest Jorden index, obtained from the ROC curve of Model 1. The cutoff points of cumulative methylation levels at all CpG sites of *VDR*, *CYP24A1, CYP27B1* and *CYP2R1* were 0.44, 1.80, 0.51 and 0.26, respectively. The methylation status of the *CYP2R1* at all CpG sites was inversely associated with colorectal cancer risk, with an adjusted OR (aOR) of 0.49 (95% CI, 0.26–0.91; *P* = 0.02) comparing the hypermethylation with hypomethylation of the *CYP2R1* gene. In contrast, no statistical association was found between the methylation status of *VDR*, *CYP24A1*, and *CYP27B1* at all CpG sites and the risk of colorectal cancer (Fig. 1).

### Association of cumulative methylation levels of significant CpG sites in *VDR*, *CYP24A1* with colorectal cancer risk

**Fig. 2:**
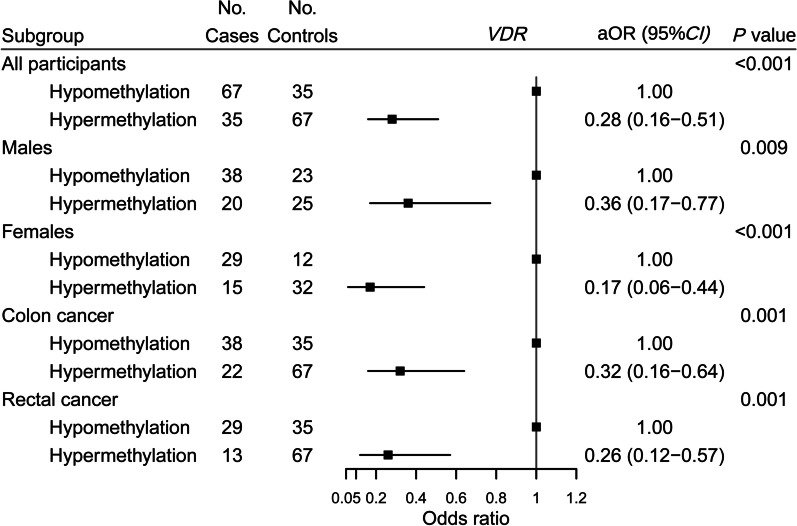
Association of cumulative methylation status at significant CpG sites of *VDR* with colorectal cancer risk. OR was adjusted for the first-degree relatives with cancer

Taking the point with the largest Youden index as the boundary value, the cumulative methylation level of *VDR* and *CYP24A1* in Model 2 was divided into hypomethylation and hypermethylation. The thresholds for cumulative methylation levels of significant CpG sites in *VDR* and *CYP24A1* were 0.066 and 0.042, respectively. The methylation status at significant CpG sites of *VDR* was negatively associated with the risk of colorectal cancer, with aOR of 0.28 (95% CI, 0.16–0.51; *P* < 0.001). Sex-stratified analysis showed that cumulative methylation status at significant CpG sites of *VDR* was significantly associated with lower risk of colorectal cancer in both males and females. However, the interaction was not significant (*P*_interaction_ = 0.26). Subgroup analysis by cancer site showed that hypermethylation of *VDR* at significant CpG sites was inversely associated with both colon cancer risk (aOR, 0.32; 95% CI, 0.16–0.64; *P* = 0.001) and rectal cancer risk (aOR, 0.26; 95% CI, 0.12–0.57; *P* = 0.001) (*P*_heterogeneity_ = 0.65) (Fig. 2).

**Fig. 3:**
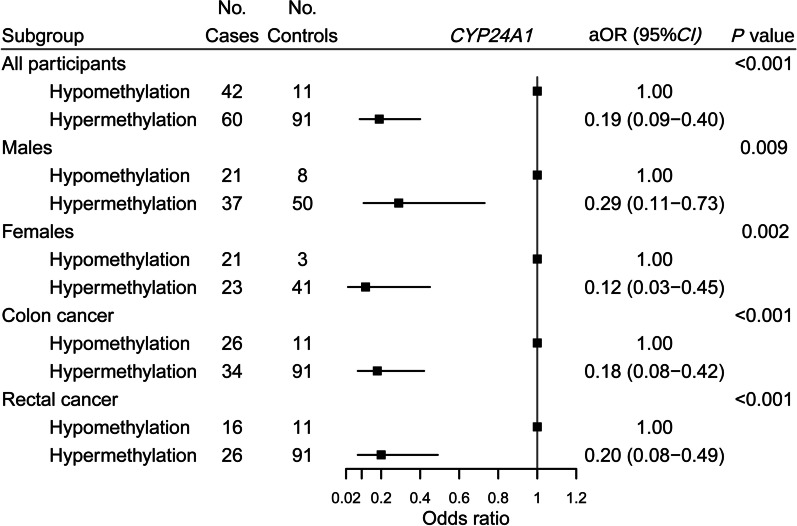
Association of cumulative methylation status of *CYP24A1* at significant CpG sites with colorectal cancer risk. OR was adjusted for the first-degree relatives with cancer

The cumulative methylation status at significant CpG sites of *CYP24A1* was negatively associated with the risk of colorectal cancer, with an aOR of 0.19 (95% CI, 0.09–0.40; *P* < 0.001). Stratified analysis by sex revealed that cumulative methylation status of *CYP24A1* at significant CpG sites was associated with a decreased risk of colorectal cancer in both males and females, which was consistent with the primary analysis (*P*_interaction_ = 0.17). According to the results of subgroup analysis of the cancer site, the hypermethylation of *CYP24A1* was inversely associated with the risk of both colon and rectal cancer, with aORs of 0.18 (95% CI, 0.08–0.42; *P* < 0.001) and 0.20 (95% CI, 0.08–0.49; *P* < 0.001), respectively (*P*_heterogeneity_ = 0.86) (Fig. 3).

## Discussion

This study aimed to use all CpG sites and significant CpG sites in each vitamin D metabolic pathway related gene to analyze cumulative methylation levels of these genes and their associations with colorectal cancer risk. The results showed an inverse association between cumulative methylation levels of all CpG sites in *CYP2R1* and colorectal cancer risk. However, there were no significant associations between cumulative methylation levels of all CpG sites in *VDR*, *CYP24A1* and *CYP27B1* and colorectal cancer risk. There were significant inverse associations between cumulative methylation levels of significant CpG sites in *VDR* and *CYP24A1* and colorectal cancer risk. No significant association was found between cumulative methylation level of significant CpG sites in *CYP2R1* and *CYP27B1* and colorectal cancer risk.

In present study, no significant association was found between cumulative methylation level of all CpG sites in *VDR* and colorectal cancer risk. However, our study showed that cumulative methylation level of significant CpG sites in *VDR* was inversely associated with colorectal cancer risk, which was inconsistent with our hypothesis. To our knowledge, no epidemiological study has reported the association between blood-based methylation level of *VDR* and colorectal cancer risk. Only one study from India including 75 colorectal cancer patients observed that the frequency of *VDR* promoter methylation in colorectal cancer tissue was significantly higher than those in normal tissues and the methylation level of *VDR* in colorectal cancer tissues was negatively correlated with its expression [22]. The inconsistency with our finding may be due to differences in genetic background among different ethnic groups. Charlene Andraos et al. found differences in the methylation levels of *VDR* between European and African races, among which African ancestry had higher methylation levels at specific CpG sites [31]. The methylation detection methods and the tissue source of the DNA samples may also partly contribute to the inconsistent results. Our study performed a next-generation sequencing approach using DNA samples from peripheral blood leukocytes, while the latter [22] was tested by Methylation-Specific PCR on DNA samples from colorectal cancer tissue. In addition, the *VDR* gene encodes the vitamin D receptor, mediating the biological effects of 1,25(OH)_2_D [32]. It was reported that vitamin D receptor plays an important role in regulating cell proliferation, differentiation and inducing apoptosis in intestinal cells [33, 34]. Our results suggest that hypomethylation of significant CpG sites in *VDR* maybe a potential biomarker of colorectal cancer. However, how hypomethylated *VDR* gene made an impact on its expression and function remains to be investigated.

Our study found that the cumulative methylation level of all CpG sites in *CYP24A1* did not differ between colorectal cancer cases and controls. It was also not significantly associated with colorectal cancer risk. However, the cumulative methylation level of significant CpG sites in *CYP24A1* was significantly lower in colorectal cancer cases than that in controls, and it was significantly negatively associated with colorectal cancer risk, which was consistent with our hypothesis*.* It seemed that cumulative methylation level in *CYP24A1* after excluding non-significant CpG sites was more representative in showing differences between the two groups. Consistent with our results, a study from Austria including 20 colorectal cancer cases reported that the methylation levels of the two regions of the *CYP24A1* promoter in cancer tissues were lower than that in normal mucosa, but the difference was not statistically significant [35]. *CYP24A1* encodes 24-hydroxylase and its high expression accelerates the inactivation of 1,25(OH)_2_D. It was reported that *CYP24A1* is overexpressed in various human tumors including colorectal cancer, and the alternation of *CYP24A1* expression is related to the development of cancer [36–38]. The expression of *CYP24A1* was in part regulated by DNA methylation, which has been observed in both lung [39] and prostate adenocarcinomas [40]. In our study, colorectal cancer cases had lower cumulative methylation level of significant sites in *CYP24A1*, suggesting that *CYP24A1* hypomethylation may contribute to the development of colorectal cancer by regulating high expression of 24-hydroxylase.

No statistically significant difference was found in our study in the methylation of single CpG site and multiple CpG sites in *CYP27B1* between the colorectal cancer cases and the controls. The *CYP27B1* gene encodes 1α-hydroxylase, which converts 25 (OH)D to the active form 1,25 (OH)_2_D. Repression of 1α-hydroxylase has been shown to be regulated by epigenetic mechanisms in a cellular experiment [41]. However, no significant association was observed between the methylation level of *CYP27B1* and colorectal cancer risk in our study. More studies are needed to explore the relationship between the methylation level of *CYP27B1* and colorectal cancer and its mechanism.

Our study found that higher cumulative methylation level of all CpG sites in *CYP2R1* was associated with a reduced risk of colorectal cancer. However, the cumulative methylation level of all CpG sites of *CYP2R1* in the colorectal cancer cases was non-significantly lower than that in controls. For *CYP2R1*, although only one CpG site was significantly different between cases and controls, there was an inverse association between cumulative methylation levels of all CpG sites in *CYP2R1* and colorectal cancer risk. It seems that the significant CpG site made great contribution for the observed association. The *CYP2R1* gene encodes the enzyme 25-hydroxylase, which converts vitamin D in the blood into the circulating form of 25(OH)D [42]. It is unclear whether such subtle methylation differences alter the effect in the pathogenesis of colorectal cancer through increased 25-hydroxylase expression. Previous study identified a significant increase in *CYP2R1* expression in renal cell carcinoma tissues [43]. It was also reported that increased regulation of the vitamin D synthesis gene *CYP2R1* and *CYP27B1* and vitamin D metabolic genes *CYP24A1* leading to changes in vitamin D bioavailability and anti-tumor activity, then influence the development of cancer [43].

Our study has some strengths. This is the first study to investigate the relationship between blood-based methylation level of multiple vitamin D metabolic pathway genes and the risk of colorectal cancer systematically. Moreover, most previous studies evaluating methylated DNA as a biomarker for colorectal cancer included fewer colorectal cancer patients (< 50 cases) [44–46]. Our study included a relatively large number of study subjects (102 colorectal cases and 102 controls) and collected comprehensive factors related to the incidence of colorectal cancer to provide epidemiological support. Additionally, in this study, MethylTarget assays based on next-generation sequencing platform were used to detect methylation levels of vitamin D metabolic pathway genes, a targeted bisulfite sequencing method which is becoming increasingly popular due to its high accuracy, flexibility and cost effectiveness.

Some limitations should be acknowledged in our study. First, as a hospital-based case–control study, selection bias could not be avoided. In our study, all colorectal cancer cases were from Sun Yat-sen University Cancer Center. However, the clinical characteristics of the patients admitted to this cancer center were similar to those of other hospitals inside [47] and outside [48] Guangdong Province. Second, methylation levels of vitamin D metabolic pathway related genes in peripheral blood leukocytes were not compared with those in colorectal tissue. Aberrant DNA methylation occurs in most colorectal cancer tissues [49, 50]; therefore, detection of methylation levels in tissues might be more accurate. However, previous studies have shown that the DNA methylation status of free cells is similar to that of primary tumor tissues [40, 51]. Ally et al. found the same trend in methylation levels of the estrogen receptor α gene in peripheral blood leukocyte DNA and colon tissue [51]. Furthermore, it is hardly possible to obtain colorectal tissue since colorectal cancer patients are at high bleeding risk. Third, there may be some measurement errors in the process of DNA methylation level detection. In order to minimize the error, all DNA samples were tested in the same batch using a blind method. To ensure the reliability of MethylTarget, previous study detected the methylation levels of *HOXA5* through Bisulfite Sequencing PCR and the results of the two methods were consistent, indicating that the results were reliable [52].

## Conclusions

In summary, this study suggested that cumulative methylation levels of vitamin D metabolic pathway related genes may be associated with colorectal cancer risk, which have not been identified in previous relevant studies. The cumulative methylation levels of significant CpG sites in *VDR* and *CYP24A1* and all CpG sites in *CYP2R1* were inversely associated with colorectal cancer risk. These results provided the scientific basis for elucidating the pathogenesis of colorectal cancer. Epigenetic alterations usually occur earlier in the early stages of colorectal cancer. Therefore, altered methylation levels in genes related to the vitamin D metabolic pathway may help to capture the early lesions of colorectal cancer. Due to the paucity of relevant studies, more research is needed to further validate these results observed in our study. Its exact mechanism is yet to be clarified.

## Data Availability

The data that support the findings of our study are available from the corresponding author upon reasonable request.

## Abbreviations

1,25(OH)_2_D: 1,25-Dihydroxyvitamin D
25(OH)D: 25-Dihydroxyvitamin D
aOR: Adjusted odds ratio
AUC: Area under the curve
BMI: Body mass index
CI: Confidence interval
MET: Metabolic equivalent
PCR: Polymerase chain reaction
ROC: Receiver operating characteristic
SD: Standard deviation
